# The perceived impact of curricular and non-curricular factors on specialty interests and choice during medical school at a single center in the United States

**DOI:** 10.1186/s12909-023-04731-1

**Published:** 2023-10-06

**Authors:** Naveen Karthik, Marjorie Greenfield, Todd Otteson

**Affiliations:** Case Western Reserve University School of Medicine, 9501 Euclid Ave, Cleveland, OH 44106 USA

**Keywords:** Specialty choice, Specialty interests, Medical education, Mentoring, Curriculum

## Abstract

**Background:**

Limited information exists regarding how medical students’ specialty interests evolve throughout medical school, particularly interest in surgical versus non-surgical specialties. Our objective was to identify medical students’ specialty interests before and after medical school and the most important curricular and non-curricular factors that shaped their specialty choice.

**Methods:**

An online 22-question voluntary, anonymized survey was designed to assess specialty interests and factors impacting specialty choice at a single medical school in the United States. The study was pilot-tested with focus groups. The final questionnaire was distributed to final-year medical students from the Classes of 2020 and 2021. Responses were measured on a 5-point Likert scale (1 = strong negative impact to 5 = strong positive impact).

**Results:**

102 of 184 students (55%) from Class of 2020 and 85 of 174 students (49%) from Class of 2021 participated. Of 187 respondents, the majority (60%) decided on their specialty during third year. 74 of 147 students (50%) pursued a specialty among their initial specialty interests. Students with initial surgical interests were significantly (p < 0.001) less likely to choose surgical specialties (42%) compared to students with initial non-surgical interests choosing non-surgical specialties (79%). Pre-clinical years (3.67 ± 0.96) were perceived to have a significantly (p < 0.001) less positive impact on specialty interests and choice compared to clinical years. Among pre-clinical factors, physician shadowing (3.80 ± 0.83) was perceived to have the significantly (p < 0.001) greatest positive impact. During clinicals, 34% of respondents indicated that order of clerkships impacted specialty choice. 112 of 171 respondents (65%) indicated that mentorship impacted specialty choice. Physicians in the chosen specialty were perceived to have the strongest impact (4.67 ± 0.49). 65 of 171 respondents (38%) indicated that peers impacted specialty choice with classmates (3.98 ± 0.87) and near-peers (3.83 ± 0.74) perceived to have a positive impact.

**Conclusions:**

Specialty interests changed during medical school for a significant portion of students (50%). Those with initial surgical interests were more likely to change their specialty interests. Pre-clinicals were reported to have less impact on specialty choice compared to clinicals. Implementing factors such as shadowing and physician/peer mentorship, which may positively impact specialty choice, into pre-clinical curricula warrants further investigation.

**Supplementary Information:**

The online version contains supplementary material available at 10.1186/s12909-023-04731-1.

## Background

Choosing a medical specialty is a pivotal decision that medical students make by their final year of medical school in the United States (U.S.). Research has shown that several factors impact specialty decisions including academic interests, lifestyle and work schedule, income, prestige, job market, student debt, board scores, and personality [1–5]. Students place varying levels of importance on these factors depending on their specialty choice as demonstrated by studies in fields including surgery, emergency medicine, psychiatry, and pathology [6–9].

Though factors related to final specialty choice have been investigated in several studies, our current understanding of how the medical school years shape the progression of specialty interests is limited. Few studies have investigated the progression of specialty interests, with wide fluctuation in the reported percentage of students (20–69%) who maintain stable specialty interests throughout medical school [10–12]. Several of these studies are limited by a focus on primary care specialties such as internal medicine, family medicine, and pediatrics. Evidence also shows that there may be a discordance in the stability of specialty interests among students who pursue primary care versus non-primary care specialties in U.S. schools [11].

Loss of interest for certain specialties over the course of medical school is a particular concern in the context of current physician workforce trends. A 2021 report by the Association of American Medical Colleges has shown significant current physician shortages in both non-surgical and surgical specialties, as recognized by the American College of Surgeons, and predictions for shortages to only worsen over the next two decades [13]. While residency applications to surgical specialties have increased in recent years, applications to non-surgical fields such as family medicine and pediatrics have declined [14]. These differences in application trends, especially in surgical versus non-surgical specialties, are important to investigate to address the most pressing workforce needs. One potential explanation for these trends may be students’ exposure and interest in specialties during medical school. Improving our knowledge of the progression of specialty interests is important for not only supporting student interests but also addressing barriers to interest and choice of specialties.

Investigating the curricular and non-curricular factors that impact specialty interests and choice is also of importance. A select few studies have examined how curricular components impact specialty interests and choice in the U.S. One study found that the pre-clinical curriculum was perceived to impact specialty choice for several students (44%) [15]. Others curricular factors that have been perceived to impact specialty choice include core clerkships (70% of students in one U.S. study) [16], clinical electives [17], and sub-internships (61% of students in one U.S. study) [18]. Furthermore, non-curricular factors may have a perceived impact on specialty interests and choice. Studies have found a strong impact of mentorship on specialty choice. In one U.S. study, exposure to a primary care physician was found to strongly predict the choice of a primary care specialty [19]. In another U.S. study, 64% of students cited mentorship having a perceived impact on specialty choice [20]. Other non-curricular factors perceived to impact specialty choice include near-peer mentorship [21] and research experience in a particular specialty [22]. Thus, non-curricular factors perceived to impact specialty choice deserve further investigation.

Understanding the progression and factors involved in specialty interests and choice may provide insight into how schools can engage students in specialty exploration, especially in fields with the greatest need for future physicians. Therefore, an objective of this study was to understand how overall specialty interests, and interest in surgical versus non-surgical specialties, evolve by comparing interests before medical school and during the final year of medical school at one center in the U.S. A second objective was to elucidate the most important curricular and non-curricular factors that shaped students’ specialty interests and final specialty choice.

## Methods

A cross-sectional survey designed to assess specialty interests through medical school and the curricular and non-curricular factors impacting specialty interests and choice was distributed to final year medical students at Case Western Reserve University (CWRU) School of Medicine.

CWRU School of Medicine is a private research university located on an urban campus in the Midwest of the United States. The structure of the University Program at CWRU School of Medicine consists of four years of medical school. The first two years are considered the “pre-clinical” years and involve classroom study of the basic and clinical sciences. The last two years are considered the “clinical” years and involve experiential learning with “clerkships” in core specialties including internal medicine, family medicine, surgery, obstetrics and gynecology, pediatrics, neurology, and psychiatry. Additionally, students may pursue elective time during the clinical years to explore other specialties. Students may also pursue an “acting internship”, also called “sub-internship”, in an interested specialty during their clinical years where they take on advanced level responsibilities at the level of a resident physician. The final year of medical school is when students decide on their specialty of choice and apply for a residency training position in that specialty.

This school has an advising system led by Academic Deans to help students with the specialty selection process. Any further mentorship in specific specialties was led by the students’ discretion. Certain students may elect to take “gap years” where they pursue further experiences to enhance their medical education such as additional degrees, research, teaching experiences, etc. Of note, the COVID-19 pandemic did cause changes with the structuring of the clinical years for the Class of 2020 and Class of 2021. Certain clinical rotations needed to be adopted into a virtual format during 2020, however most rotations returned to in-person by 2021.

In order to capture the most relevant factors that influenced students’ specialty interests and choice, focus groups were conducted with final year medical students. In these focus groups, we interviewed seven final year medical students and collected their perspectives on how they arrived at their choice of specialty. Students listed curricular and non-curricular factors that impacted their specialty choice. They also shared the time point at which they decided on their specialty and why they made the decision at that time. The authors compiled these narratives from the focus groups and used content derived from previously validated and published surveys on specialty interests and choice [6–11, 23, 24] to create a 22-question survey. The survey was designed using the Qualtrics Survey Software (Qualtrics, Seattle, WA), which also collected and compiled the responses.

The survey was distributed to all final-year medical students from two consecutive medical school classes (Class of 2020 and Class of 2021). This study was exempt from IRB review by CWRU School of Medicine’s IRB committee (STUDY20191713 approved on February 23rd, 2021). An initial email with an invitation to the study was shared with students and then a follow-up reminder email was sent. Students were informed in all correspondence that participation in the survey was voluntary, all responses would be anonymized, and that participation would not impact their academic standing. For the Class of 2020, the survey form was accessible from February 2020 to April 2020. For the Class of 2021, the survey form was accessible from November 2020 to December 2020. Participants were surveyed about their chosen medical specialty, year of medical school they chose that specialty, and the main reason(s) for their specialty choice. Participants were then asked about specialty interests before medical school, including a general interest in surgery and interest in specific specialties. Specialties were categorized as surgical and non-surgical for the purpose of analysis. Surgical specialties included those recognized by the American College of Surgeons [25], which in this study were cardiac surgery, general surgery, obstetrics and gynecology, neurological surgery, ophthalmology, orthopaedic surgery, otolaryngology, plastic surgery, urology, and vascular surgery. All other specialties were categorized as non-surgical.

The perceived impact of curricular factors on specialty interests and choice was determined using a 5-point Likert scale (1 = strong negative impact, 2 = moderate negative impact, 3 = no impact, 4 = moderative positive impact, 5 = strong positive impact). Participants were asked if exposure to specialties of interest during different time periods of medical school (pre-clinical years, core clerkships, elective rotations, acting internships, rotations outside the home institution, and gap year(s) during medical school) had a perceived impact on their specialty interests and choice. The impact of components of the pre-clinical years (classroom subject material, events/keystone lectures, interest groups, and physician shadowing) on specialty interests and choice was also queried. Lastly, participants were asked if they believed that the order of clerkships had a perceived impact on their specialty choice and to answer through a free response prompt in what way the order had an impact. Lastly, the perceived impact of non-curricular factors was also investigated using the same 5-point Likert scale. Participants were asked about the impact of mentorship, peers, home residency program, and research on specialty interests and choice.

All responses were compiled and downloaded from Qualtrics. Responses were collected from participants regardless of whether the participant completed the entire survey. Any questions not answered by the participant were omitted from the analysis. Statistics including one-way ANOVA with post-hoc analysis using Tukey’s honest significance test, Student’s t-tests, chi-square tests, and descriptive analytics of the progression of specialty interests and the impact of curricular and non-curricular factors on specialty interests and choice were performed using Minitab (State College, PA). P ≤ 0.05 was considered statistically significant.

## Results

**Fig. 1:**
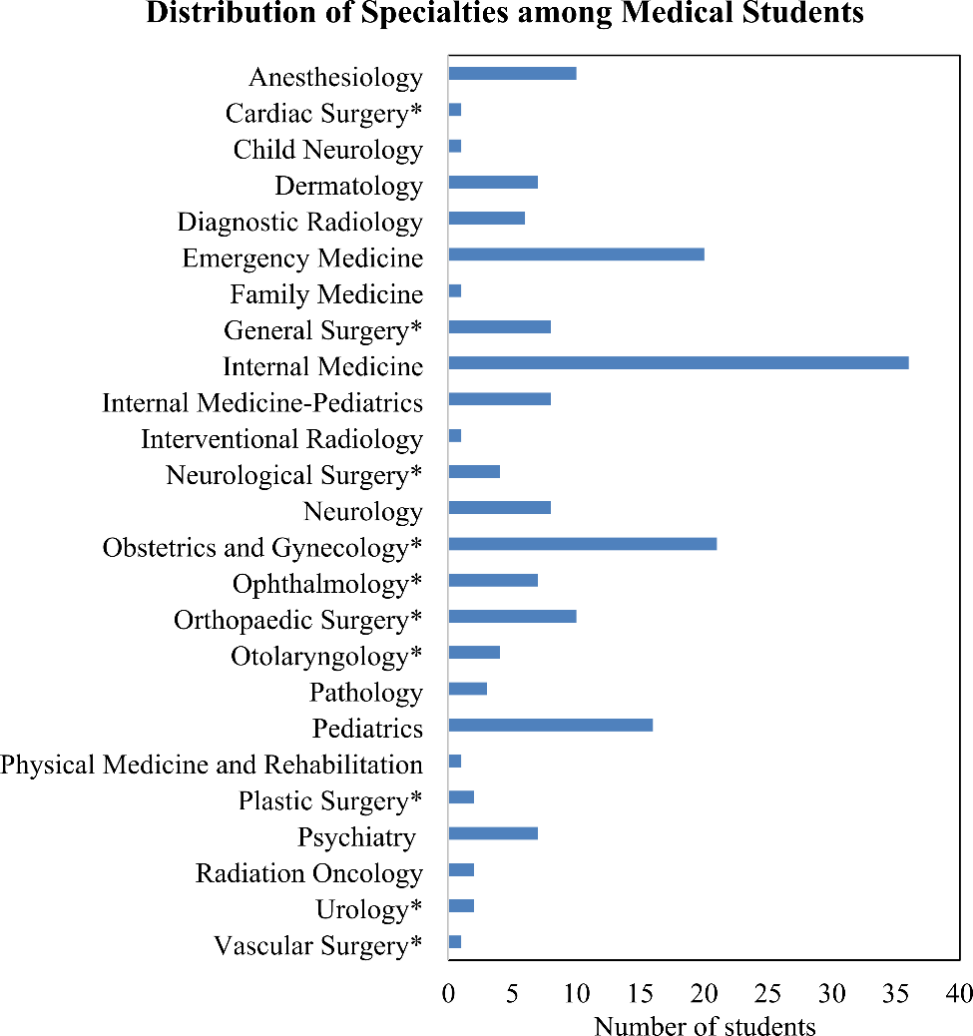
Distribution of specialty choices (surgical and non-surgical) among survey respondents from two graduating classes (Class of 2020 and Class of 2021) at one institution. *Surgical specialties recognized by the American College of Surgeons [25]

Of the 184 students in the Class of 2020, 102 students chose to participate (55%) and of the 174 students in the Class of 2021, 85 students chose to participate (49%). Figure 1 shows the distribution of chosen specialties from the 187 respondents. 112 of 187 respondents (60%) decided on their specialty during the third year of medical school, while 26 (14%) decided during first year, 16 (9%) decided during second year, 24 (13%) decided during fourth year, and 10 (5%) decided during gap year(s) during medical school. The most commonly cited reasons for choosing a specialty included the scope of practice and clinical variety (n = 63), subject matter of the specialty (n = 54), and the patient population (n = 46) as shown in Table 1.

**Table 1:** Most Common Reasons for Specialty Choice

Reason for choice of specialty	Number of Students
Scope of practice and clinical variety	63
Subject matter	54
Patient population	54
Culture/fit of the profession	46
Ability to specialize or tailor career	31
Lifestyle (work-life balance)	31
Mentors	16
Interesting research opportunities	16
Clinical rotations/electives	16
Mix of surgery and patient care	15

147 of 187 students (79%) reported specialty interests before medical school, of which 74 (50%) pursued a specialty among their initial specialty interests. 70 of 147 students (48%) reported interest in surgery before medical school, 62 (42%) reported no interest in surgery, and 15 (10%) were unsure about their interest in surgery. Among the 70 students interested in surgery initially, 30 (42%) ultimately pursued a surgical specialty. Of the 62 students initially not interested in surgery, 49 (79%) pursued a non-surgical specialty. Students initially interested in surgery were significantly (χ2 = 17.91, p < 0.001) less likely to maintain their initial interest compared to students initially not interested in surgery.

Table 2 shows the perceived impact of curricular factors on specialty interests and choice. There was a statistically significant difference in mean Likert ratings between time periods in medical school (F(5, 1068) = 68.0, p < 0.001). Pre-clinical years were perceived to have less of a positive impact on specialty interests and choice compared to acting internships, electives, or core clerkships. Table 3 shows the impact of pre-clinical curricular factors on specialty interests and choice. Shadowing with physicians was perceived to have the greatest positive impact compared to other components of the pre-clinical curriculum.

**Table 2:** Perceived Impact of Curricular Factors on Specialty Interests and Choice

Curricular Factor	Mean Likert Scale Rating of Importance: 1 = strong negative impact to 5 = strong positive impact	Standard Deviation
Acting internship	4.48	0.81
Elective rotation	4.30	0.83
Core clerkships	4.29	1.01
Pre-clinical years*	3.67	0.96
Rotations outside the home institution	3.38	0.75
Gap year(s) during medical school	3.29	0.70

*Pre-clinical years were reported to have a significantly (p < 0.001) lower mean Likert Scale rating for perceived impact on specialty interests and choice compared to acting internships, elective rotations, and core clerkships

**Table 3:** Perceived Impact of Pre-clinical Curriculum on Specialty Interests and Choice

Pre-clinical Curricular Factor	Mean Likert Scale Rating of Importance: 1 = strong negative impact to 5 = strong positive impact	Standard Deviation
Shadowing with physicians*	3.80	0.83
Classroom subject material related to chosen specialty	3.44	0.78
Events/keystone lectures in chosen specialty	3.42	0.65
Involvement in interest groups	3.38	0.70

*Shadowing with physicians was reported to have a significantly (p < 0.001) higher mean Likert Scale rating for perceived impact on specialty interests and choice compared to classroom subject material, events/keystone lectures, and involvement in interest groups

During the clinical years, 60 of 177 (34%) respondents indicated that order of clerkships impacted specialty choice. Among responses from these 60 students, the most commonly cited reasons for how the order of clerkships perceived to impact specialty choice included the following: (1) Students chose a specialty because they had a rotation in that specialty early in the year and had more time to make a decision (n = 5), (2) Students did not pursue a specialty of interest due to late exposure and not enough time to plan acting internships/applications (n = 5), (3) Having opportunities for surgical electives early during clinical rotations helped in the decision to choose surgery (n = 3), and (4) Having the chosen specialty last allowed the student to rule out other specialties before making their decision (n = 3).

112 of 172 respondents (65%) perceived that mentorship impacted specialty interests and choice. As shown in Table 4, physicians in the chosen specialty were perceived to have the greatest positive impact on specialty interests and choice compared to other mentors. 65 of 172 respondents (38%) reported that peers impacted specialty interests and choice. Table 4 shows that classmates were perceived to have a greater positive impact on specialty interests and choice compared to peer(s) or friend(s) outside of medical school, but no difference was noted in perceived positive impact between classmates and near-peers. 165 of 167 students (99%) reported having access to a home residency program in their chosen specialty. Table 4 shows that research and shadowing opportunities through a home residency program were reported to have a greater positive perceived impact on specialty interests and choice compared to networking events.

**Table 4:** Perceived Impact of Non-Curricular Factors on Specialty Interests and Choice

Non-Curricular Factors		Mean Likert Scale Rating of Importance (1 = strong negative impact to 5 = strong positive impact)	Standard Deviation
Mentorship			
	Physician in chosen specialty^a^	4.67	0.49
	Physician not in chosen specialty	3.43	0.91
	Academic Dean	3.36	0.73
	Non-physician	3.31	0.66
Peers			
	Classmates^b^	3.98	0.87
	Near-peer mentor	3.83	0.74
	Peer or friend outside of medical school	3.54	0.75
Interactions with Home Residency Program			
	Research opportunities^c^	3.78	0.88
	Shadowing opportunities^c^	3.74	0.78
	Networking events	3.51	0.71

^a^Physician in chosen specialty was perceived to have the significantly (p < 0.001) highest mean Likert Scale rating for impact on specialty interests and choice compared to physician not in chosen specialty, Academic Dean, and non-physician

^b^Classmates were perceived to have a significantly (p < 0.01) higher mean Likert Scale rating for impact on specialty interests and choice compared to peer(s) or friend(s) outside of medical school

^c^Research and shadowing opportunities were perceived to have significantly (p < 0.05) higher mean Likert Scale ratings for impact on specialty interests and choice compared to networking events

158 of 167 (95%) students in this study reported being involved in a research project in medical school. 118 of 158 (75%) students reported pursuing research in their chosen specialty. Of the 158 students who pursued research, 38 students (24%) perceived a strong positive impact of research on specialty interests and choice, 60 students (38%) perceived a moderate positive impact, and 56 students (35%) perceived no impact.

## Discussion

The progression of specialty interests throughout medical school and the curricular and non-curricular factors that affect specialty interests and choice are areas that have needed further research. Results from this study suggest that specialty interests change for many students, especially for students who enter medical school with surgical interests. Students may perceive that curricular factors, including core clerkships, acting internships, and electives, along with non-curricular factors, including mentorship, peers, access to a home residency program, and research, may positively impact their specialty interests and final choice.

Several students in this study (50%) reported initial specialty interests that were different from their final specialty choice. Past studies investigating the progression of specialty interests have shown inconsistent findings. One national study found only 30% of students maintained an interest in primary care specialties across medical school, while 68% maintained an interest in non-primary care specialties throughout medical school [11]. Another study, however, found that the stability of specialty interests was quite low (20%) and that specialty interests were more stable across medical school for generalist specialties such as family practice, internal medicine, and pediatrics and were less stable for other non-primary care specialties [10]. In our study, students with initial non-surgical interests were more likely to maintain their decision (79%) throughout medical school compared to students who were initially interested in surgery, among whom only 42% went on to pursue a surgical specialty.

Students with initial surgical interests may be less likely to ultimately choose a surgical specialty for several potential reasons. One of these reasons is the amount of surgical exposure. One U.S. study found that amount of operative experience and interaction with residents and faculty was perceived to strongly impact students who had increased interest in surgery after their clerkship [26]. Another factor may be the perceived stereotypes of surgical professions and the perceived competitiveness of obtaining a surgical residency. In a qualitative study conducted in the United Kingdom, students revealed factors that dissuaded them from a career in surgery including the gender stereotypes and perceived competitiveness of surgical professions [27]. Our results along with findings from the literature suggest that medical students who are interested in surgery would benefit from active engagement in the field including having early exposure, positive role models, and discussions about negative stereotypes in the field.

Curricular factors may also have a perceived impact on specialty interests and choice. The pre-clinical years were perceived as the curricular time period with the least positive impact on specialty interests and choice. During pre-clinical years, shadowing with physicians was perceived to have the most positive impact on specialty interests and choice. Previous efforts integrating shadowing and clinical skills programs into pre-clinical years have shown to be effective in promoting early interest in specialty decision-making [28–32]. Primary care experiential learning during the first year of medical school has been positively associated with a student’s choice of primary care specialties [29], which is particularly important due to the recent trend in declining applications to non-surgical fields [14]. Pre-clinical surgical electives have also shown success in influencing a student’s decision to pursue a surgical career [31], an important consideration for students entering medical school with an interest in surgery.

Most students in this study (60%) chose their specialty during their third year of medical school, and many (34%) perceived that the order of clerkships impacted their specialty choice. Few studies have investigated the impact of clerkship order on specialty choice. One study found that scheduling clerkships to students’ preferences may make students more likely to place rotations for specialties of interest in the middle blocks of the academic year [33]. Positioning these rotations as such may be beneficial as students are provided time to acclimate to the rigorous clerkship environment in the early blocks while still allowing time to plan for electives and acting internships during later blocks of the year. In this study, five students chose not to pursue a specialty of interest due to late exposure and not enough time to plan acting internships/applications. A preference-based clerkship experience may help address this type of barrier. However, limitations exist with preference-based scheduling of clerkships including increased competition for certain clerkships and decreased enrollment in other clerkships during particular times of the year, which may present administrative challenges [33]. Benefits of preference-based ordering of clerkships and electives, particularly for students interested in specialties underrepresented in core clerkships, require further investigation.

Acting internships and elective rotations were also perceived to have a strong positive impact on specialty interests and choice. Previous studies have shown that acting internships, also known as sub-internships, have a positive reported impact on specialty choice, especially in surgical specialties in which there may be limited exposure during core clerkships [18]. In specialties without acting internships, electives have been shown to be effective in increasing positive perceptions and interest in these specialties [34, 35]. These findings demonstrate that for students who are interested in specialties with limited representation in the core clerkships, early involvement in electives and pursuit of acting internships could be encouraged to foster interest in those professions.

Several non-curricular factors were also perceived to impact specialty interests and choice. Among mentors, physicians in students’ chosen specialty were perceived to have the most positive impact on specialty interests and choice. Several studies have demonstrated the benefits of mentorship on choosing a specialty, particularly for students who express early interest in primary care and surgical fields [19, 22, 36–37]. Female students, students from minority groups underrepresented in medicine (URM), and students who are first generation college graduates (FG) have historically lacked quality and quantity of mentorship early in their medical careers [20, 38]. For these individuals, having role models and mentors from shared backgrounds can greatly increase their interest in fields such as surgery, where they are traditionally underrepresented [34, 39]. Likewise, near-peers may also have a role in providing mentorship to students. Near-peer mentorship pilot programs have been successful in cultivating career support and specialty exploration, particularly among URM students and FG students who may otherwise not have access to physician role models [21]. Among peer groups in this study, near-peers were perceived to have a positive perceived impact on specialty interests and choice. Thus, integrating physician and near-peer mentorship programs may be a consideration in curricular design.

Almost all students (99%) in this study reported having a home residency program in their chosen specialty. Residents can be effective clinical teachers and role models for students and may have a positive impact on specialty choice [40]. In this study, research and shadowing with the home residency program were perceived to have the most positive impact on specialty interests and choice. Research experiences have been shown to increase specialty interests over the course of medical school, especially in surgery [22]. A majority of students (62%) in this study perceived that research had at least a moderately positive impact on specialty choice. These results suggest that connection with a home residency program in specialties of interest for resident mentorship, shadowing, and research opportunities could be encouraged early on in medical school. Furthermore, students interested in more competitive specialties may benefit from research and department connections for strengthening their applications. For students without a home residency program in specialties of interest, programs may consider connecting these students with physician mentors in the community to increase exposure.

There were several limitations to the present study. This was a single institution study and was limited to two graduating classes. Specialty choice distributions vary across medical schools and different countries due to unique school philosophies and admissions criteria potentially affecting the generalizability of these results. Also, curricular structure such as the ordering of clerkships and rotations and non-curricular factors such as access to home residency programs and research opportunities differ across institutions.

With regards to methodology, this was a survey-based study that may be affected by non-response bias. Demographic factors including age and gender were not collected to preserve anonymity. Data collection in this study occurred prior to and during the COVID-19 pandemic. Thus, the COVID-19 pandemic may serve as a confounding factor in students’ specialty interests and choice. The need for using more objective measures to check normality assumptions in this dataset is also a limitation.

Due to the limited number of questions, the scope of this survey is not able to explore all factors involved in specialty interests and choice. Though questions on this survey were primarily adopted from previously validated surveys on specialty choice, this survey still contains unique questions that were derived from investigation using focus groups. Due to the novelty of the survey in this regard, the reliability and validity of these results may be a limitation. Lastly, since this study measured perceptions of the impact of curricular and non-curricular factors on specialty interests and choice, it may have been affected by recall bias.

## Conclusions

In this study, specialty interests evolved for a significant portion of medical students through the course of medical school. Students with initial surgical specialty interests were more likely to change specialty interests during medical school compared to students with initial non-surgical interests. The pre-clinical years were perceived to have a less positive impact on specialty interests and choice compared to the clinical years but may be an opportune time to increase specialty exposure through physician shadowing, mentorship programs with physicians and near-peers, and connections with a home residency program, all factors perceived to have a positive impact on specialty choice. During the clinical years, the order of clerkships may have an important role in specialty choice, especially for planning acting internships and electives. Future work investigating the impact of clerkship order on specialty choice is warranted. Though choosing a specialty can be stressful and at times overwhelming for a medical student, targeted curricular and non-curricular efforts can facilitate specialty exploration and promote informed decision-making.

### Electronic supplementary material

Below is the link to the electronic supplementary material.

Supplementary Material 1

## Data Availability

The datasets used and/or analyzed during the current study are available from the corresponding author on reasonable request.

## Abbreviations

CWRU: Case Western Reserve University
URM: Underrepresented in medicine
FG: First generation college graduates
