# Supplementary material for: The perceived impact of curricular and non-curricular factors on specialty interests and choice during medical school at a single center in the United States
Source: BMC Med Educ. 2023 Oct 6;23:730. doi: 10.1186/s12909-023-04731-1 (PMC10559574; doi:10.1186/s12909-023-04731-1)
Supplement: Supplementary file 1 — Supplementary Material 1 [file 12909_2023_4731_MOESM1_ESM.pdf]

Demographic Information

Thank you for participating in this survey about your medical specialty decision-making process. The purpose of this study is to understand the factors that are most important to students when selecting their specialty.

Your responses will be recorded anonymously and kept confidential. Please note that participation is voluntary and that completion of any part of this survey implies your consent to participate in this study.

Please use the arrow below to begin the survey.

Medical Specialty Information

What is your chosen specialty?

- ◯ Anesthesiology
- ◯ Dermatology
- ◯ Diagnostic Radiology
- ◯ Emergency Medicine
- ◯ Family Medicine
- ◯ General Surgery
- ◯ Internal Medicine
- ◯ Medical Genetics
- ◯ Neurology
- ◯ Neurological Surgery
- ◯ Nuclear Medicine
- ◯ Obstetrics and Gynecology
- ◯ Ophthalmology
- ◯ Oral and Maxillofacial Surgery
- ◯ Orthopaedic Surgery
- ◯ Otolaryngology
- ◯ Pathology
- ◯ Pediatrics
- ◯ Physical Medicine and Rehabilitation
- ◯ Preventive Medicine
- ◯ Psychiatry
- ◯ Radiation Oncology
- ◯ Urology
- ◯ Vascular Surgery
- ◯ Other ____________

What year of medical school did you decide on your chosen specialty?

- ◯ M1
- ◯ M2
- ◯ M3
- ◯ M4
- ◯ Gap year(s) during medical school

Please briefly state the main reason(s) for your decision to pursue this specialty:

____

Block 7

Specialty Interests before Medical School

Did you have any interest(s) in particular specialties before medical school?

- ○ Yes
- ○ No

Did you have an interest in surgery before medical school?

- ○ Yes
- ○ No
- ○ Unsure

Please select the specialty or specialties that interested you before medical school (You can select more than one):

- ☐ Anesthesiology
- ☐ Dermatology
- ☐ Diagnostic Radiology
- ☐ Emergency Medicine
- ☐ Family Medicine
- ☐ General Surgery
- ☐ Internal Medicine
- ☐ Medical Genetics
- ☐ Neurology
- ☐ Neurological Surgery
- ☐ Nuclear Medicine
- ☐ Obstetrics and Gynecology
- ☐ Ophthalmology
- ☐ Oral and Maxillofacial Surgery
- ☐ Orthopaedic Surgery
- ☐ Otolaryngology
- ☐ Pathology
- ☐ Pediatrics
- ☐ Physical Medicine and Rehabilitation
- ☐ Preventive Medicine
- ☐ Psychiatry
- ☐ Radiation Oncology
- ☐ Urology
- ☐ Vascular Surgery
- ☐ Other ______

Block 6

General Information

What field was your undergraduate degree (e.g. Psychology, Biology, Comparative Literature, etc.)?

______

Did exposure to your chosen specialty during any of the following time periods have an impact on your decision to choose that specialty?

	Strong Negative Impact	Moderate negative impact	No impact	Moderate positive impact	Strong positive impact
Pre-clinical years	○	○	○	○	○
Core clerkships	○	○	○	○	○
Elective rotations	○	○	○	○	○
Acting internship	○	○	○	○	○
Away rotation(s)	○	○	○	○	○
Gap year(s) during medical school	○	○	○	○	○

Pre-clinical and Clinical Curriculum

Pre-Clinical and Clinical Years

Did any of the following factors during your pre-clinical years have an impact on your choice of specialty?

	Strong negative impact	Moderate negative impact	No impact	Moderate positive impact	Strong positive impact
Classroom subject material related to chosen specialty	◯	◯	◯	◯	◯
Events/keystone lectures in chosen specialty	◯	◯	◯	◯	◯
Involvement in interest groups	◯	◯	◯	◯	◯
Shadowing with physicians	◯	◯	◯	◯	◯

Did the order of your core clerkships have an impact on your choice of specialty?

◯ Yes

◯ No

Please briefly explain how the order of your core clerkships impacted your choice of specialty:

Mentorship and Peer Interactions

Mentorship and Peer Interactions

Did you have mentor(s) or advisor(s) that influenced your decision to pursue your chosen specialty?

◯ Yes

◯ No

Did any of the following individuals impact your decision to pursue your chosen specialty?

	Strong Negative Impact	Moderate Negative Impact	No impact	Moderate Positive Impact	Strong Positive Impact
Academic dean	◯	◯	◯	◯	◯
Physician(s) in your chosen specialty	◯	◯	◯	◯	◯
Physician(s) not in your chosen specialty	◯	◯	◯	◯	◯
Physician(s) from your medical school and/or its affiliated hospitals	◯	◯	◯	◯	◯
Physician(s) from outside your medical school and/or its affiliated hospitals	◯	◯	◯	◯	◯
Non-physician	◯	◯	◯	◯	◯

Did peers have any impact on your choice of specialty?

◯ Yes

◯ No

Did any of the following peer interactions have an impact on your choice of specialty?

	Strong Negative Impact	Moderate Negative Impact	No Impact	Moderate Positive Impact	Strong Positive Impact
Near-peer mentor (peer in medical school who is at least one year above you that provided mentorship)	◯	◯	◯	◯	◯
Classmates	◯	◯	◯	◯	◯
Peer(s) or friend(s) outside of medical school	◯	◯	◯	◯	◯

Access to Home Residency Program

Home Institution Residency Program and Research

Did your home institution have a residency program in your chosen specialty?

◯ Yes

◯ No

Did any of the following interactions with your home institution residency program have an impact on your choice of specialty?

	Strong Negative Impact	Moderate Negative Impact	No Impact	Moderate Positive Impact	Strong Positive Impact
Networking events	◯	◯	◯	◯	◯
Research opportunities	◯	◯	◯	◯	◯
Shadowing opportunities	◯	◯	◯	◯	◯

How did participating in research impact your decision to pursue your chosen specialty?

◯ Did Not Participate in Research

◯ Strong Negative Impact

◯ Moderate Negative Impact

◯ No Impact

◯ Moderate Positive Impact

◯ Strong Positive Impact

Did you pursue research in your chosen specialty?

◯ Yes

◯ No

Block 6

Other Factors Impacting Specialty Choice

Please use the drag and drop feature to rank the following factors based on their level of impact on your choice of specialty (#1 = most impactful to #9 = least impactful):

- Earning potential of your chosen specialty
- Ethnic diversity in your chosen specialty
- Geographic and/or family restrictions
- Gender representation in your chosen specialty
- Intellectual curiosity for subject matter of chosen specialty
- Job satisfaction involved with your chosen specialty
- Maintaining a work-life balance
- Perceived prestige of your chosen specialty
- Personality or culture fit of your chosen specialty

Block 5

Experiences Before Medical School

How did the following factors before medical school impact your decision to pursue your chosen specialty?

	Strong negative impact	Moderate negative impact	No impact	Moderate positive impact	Strong positive impact
Major(s)/minor(s) during undergraduate study	○	○	○	○	○
Clubs and activities during undergraduate study	○	○	○	○	○
Hospital volunteering work during undergraduate study	○	○	○	○	○
Experience as an Emergency Medical Technician (EMT)	○	○	○	○	○
Research experiences during undergraduate study	○	○	○	○	○

Did any of the following factors have an impact on your choice of specialty?

	Strong negative impact	Moderate negative impact	No impact	Moderate positive impact	Strong positive impact	Not applicable
Family or personal socioeconomic status	○	○	○	○	○	○
Family members in your chosen specialty	○	○	○	○	○	○
Family members in a surgical specialty	○	○	○	○	○	○
Family members in a non-surgical specialty	○	○	○	○	○	○
